# Microcystic Inner Nuclear Layer Changes and Retinal Nerve Fiber Layer Defects in Eyes with Glaucoma

**DOI:** 10.1371/journal.pone.0130175

**Published:** 2015-06-12

**Authors:** Tomoko Hasegawa, Tadamichi Akagi, Munemitsu Yoshikawa, Kenji Suda, Hiroshi Yamada, Yugo Kimura, Hideo Nakanishi, Masahiro Miyake, Noriyuki Unoki, Hanako Ohashi Ikeda, Nagahisa Yoshimura

**Affiliations:** Department of Ophthalmology and Visual Sciences, Kyoto University Graduate School of Medicine, Kyoto, Kyoto, Japan; Charité University Medicine Berlin, GERMANY

## Abstract

**Objective:**

To examine microcystic inner nuclear layer (INL) changes in glaucomatous eyes and to determine associated factors.

**Design:**

Retrospective, cross-sectional, observational study.

**Methods:**

Two hundred seventeen eyes of 133 patients with primary open angle glaucoma (POAG), 41 eyes of 32 patients with preperimetric glaucoma and 181 normal eyes of 117 subjects were ultimately included. Microcystic INL lesions were examined with infrared fundus images and with 19 vertical spectral domain optical coherence tomography (SD-OCT) images in the macular area.

**Results:**

Microcystic INL changes were observed in 6.0% of eyes with POAG, but none of the normal eyes or eyes with preperimetric glaucoma showed microcystic INL changes. The proportion of eyes with advanced glaucoma was significantly larger (*P* = 0.013) in eyes with microcystic lesions than without. The visual field mean deviation (MD) slope was also significantly worse (*P* = 0.027) in eyes with microcystic lesions. No significant differences were observed in age, sex, refraction, axial length, intraocular pressure, or MD value between eyes with and without microcystic INL lesions. In several cases, microcystic INL lesions occurred along with glaucomatous visual field progression. The retinal nerve fiber layer (RNFL) thickness (*P* = 0.013) and ganglion cell layer (GCL) + inner plexiform layer thickness (*P* = 0.023) were significantly lower in areas with microcystic lesions than without. The INL was also significantly thicker (*P* = 0.002) in areas with microcystic lesions.

**Conclusions:**

Microcystic INL lesions in glaucomatous eyes are closely associated with RNFL and GCL thinning and correlated with worse MD slope. These INL lesions may indicate focal and progressive damage in glaucoma.

## Introduction

Glaucoma is a leading cause of blindness throughout the world. It is a progressive optic neuropathy, resulting from axonal damage within the optic disc. This subsequently causes retinal nerve fiber layer (RNFL) and retinal ganglion cell layer (GCL) thinning [1–10].

Other retinal layers, including the inner nuclear layer (INL) and outer nuclear layer (ONL), are thought to be less affected by glaucoma, but only a few studies have examined this possibility [11–17]. Spectral-domain optical coherence tomography (SD-OCT) enables the rapid acquisition of high axial resolution retinal images, making it possible to analyze individual retinal layer changes in vivo [3–10]. It was recently shown that microcystic changes in the INL may occur in several types of optic nerve diseases, including multiple sclerosis (MS) [18–21], neuromyelitis optica (NMO) [22–24], idiopathic optic atrophy [19], relapsing isolated optic neuritis [21], comprehensive neuropathy [25], hereditary optic neuropathy [19, 26], and trauma [19]. Additionally, microcystic INL changes have been shown to be related to MS severity and recurrence rates [18, 27]. Microcystic INL changes have been previously observed in glaucomatous eyes [19], but their features have not been well characterized.

Macular edema, typically resulting from blood-retinal barrier breakdown [28–30], can be caused by many different conditions, including diabetes, uveitis, retinal vein occlusion, and age-related macular degeneration. Macular edema generally presents as focal leakage, diffuse leakage, or pooling of fluorescein, but microcystic INL lesions related to optic nerve diseases have not been associated with fluorescein leakage [19, 26]. On the other hand, intraretinal cystoid spaces without fluorescein leakage can occur in eyes with epiretinal membrane (ERM), vitreomacular traction syndrome, and myopic traction maculopathy. In these conditions, cystoid spaces are closely related to preretinal structures (e.g., partial posterior vitreous detachments [PVD] with vitreomacular traction, cortical vitreous layer remnants after PVD, and ERM) and intrinsic retinal properties (e.g., internal limiting membrane [ILM] and retinal arteriole stiffness) [31–33]. The precise mechanism by which microcystic INL changes develop in optic nerve diseases is not understood, but several potential causes have been suggested, including retrograde transcellular degeneration, Muller cell dysfunction, vitreous traction, and inflammation-induced blood-retinal barrier disruptions [18, 21, 25–27, 34].

In this study, we thoroughly analyzed retinal layers in the macula in eyes with glaucoma. We specifically examined features of and associated factors for microcystic INL changes in glaucomatous eyes. Possible mechanisms of development are also discussed.

## Methods

### Subjects

All investigations adhered to the tenets of the Declaration of Helsinki, and the study was approved by the institutional review board and the ethics committee at Kyoto University Graduate School of Medicine. The nature of the study and its possible consequences were explained to study candidates, after which written informed consent was obtained from all who participated.

**Fig 1:**
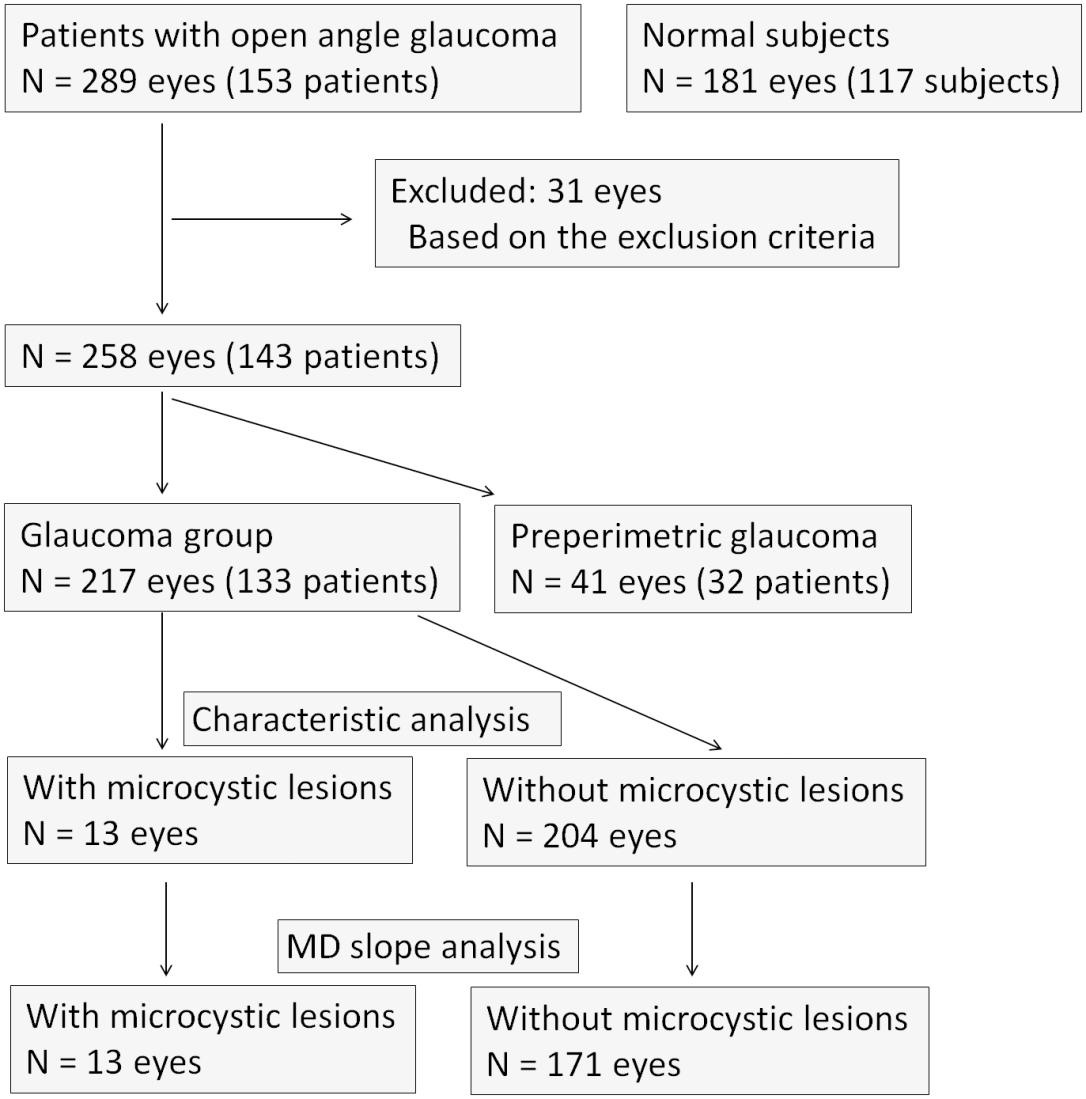
Flowchart of the subjects.

Patients with a glaucoma diagnosis who visited the Glaucoma Service at Kyoto University Hospital between April 2013 and July 2013 were considered for inclusion in this study. Normal subjects who visited our service between Feb 2011 and July 2013 were also included as normal controls (Fig 1). All patients in the database had undergone a comprehensive ophthalmic examination as a routine set of examinations in our service, including best-corrected visual acuity (BCVA) measurement (5 m Landolt chart), refraction, keratometry, slit-lamp examination, axial length measurement (IOLMaster; Carl Zeiss Meditec, Dublin, CA), Goldmann applanation tonometry, gonioscopy, indirect ophthalmoscopy, dilated slit-lamp optic nerve head examination, fundus photography, stereo disc photography (3-Dx simultaneous stereo disc camera; Nidek, Gamagori, Japan), red-free fundus photography (Heidelberg Retina Angiography [HRA] 2; Heidelberg Engineering, Heidelberg, Germany), standard automated perimetry (SAP, 24–2 Swedish interactive threshold algorithm standard program; Humphrey Visual Field Analyzer [HFA], Carl Zeiss Meditec) and imaging with SD-OCT (Spectralis HRA+OCT, Heidelberg Engineering).

Glaucoma diagnoses were made based on the presence of diffuse or localized rim thinning (determined with stereo disc photography) and/or retinal nerve fiber layer defects (NFLD) corresponding to glaucomatous visual field defects (determined with red-free fundus imaging). Optic disc appearance was independently evaluated by 3 glaucoma specialists (TA, HOI, HN) who were masked to all other patient and ocular information. If all 3 examiners were not in agreement with the ocular classification, the group re-reviewed and discussed fundus color and stereo photograph findings until a consensus was reached. Glaucoma stages were determined to be early, moderate, or advanced and were based on HFA visual field findings. Based on Hoddap-Parrish-Anderson criteria, stages were determined with MD value, number of decreased pattern deviation points and points within the central 5 degrees of vision with a sensitivity <15 dB [35]. Eyes with a normal visual field were classified into either the normal, ocular hypertension or preperimetric glaucoma group, based on disc appearance and the intraocular pressure (IOP). The HFA mean deviation (MD) slope was used to analyze the rate of visual field defect progression. Eyes with at least 4 reliable HFA examinations and 2 or more years of follow-up were included in analyses of MD slope (dB/year) (Fig 1), which utilized automated linear progression analysis (Nidek Advanced Vision Information System [NAVIS], Nidek). IOP analyses were performed with values obtained by averaging IOP measurements made at the first visit included in this study and the 4 prior consecutive visits.

Patients with a normal anterior chamber on slit-lamp examination and a normal open angle on gonioscopy were included in analyses. Patients were excluded from analyses if BCVA was worse than 20/40 (Snellen equivalent), refractive cylindrical error was more than 3.00 D, SAP visual field results were unreliable (fixation loss >20%, false-positive >15%, or false-negative >33%), or evidence of vitreoretinal disease (e.g., ERM, vitreoretinal traction syndrome, and posterior staphyloma or chorioretinal atrophy with high myopia) was present. Ocular hypertension patients were also excluded from the analysis. Patients with diabetes mellitus or any other systemic disease known to affect the eye were also excluded (Fig 1).

Preperimetric glaucoma eyes were excluded from the analysis of the characteristics between eyes with or without INL microcystic lesions (Fig 1).

### Imaging and Image Analysis

**Fig 2:**
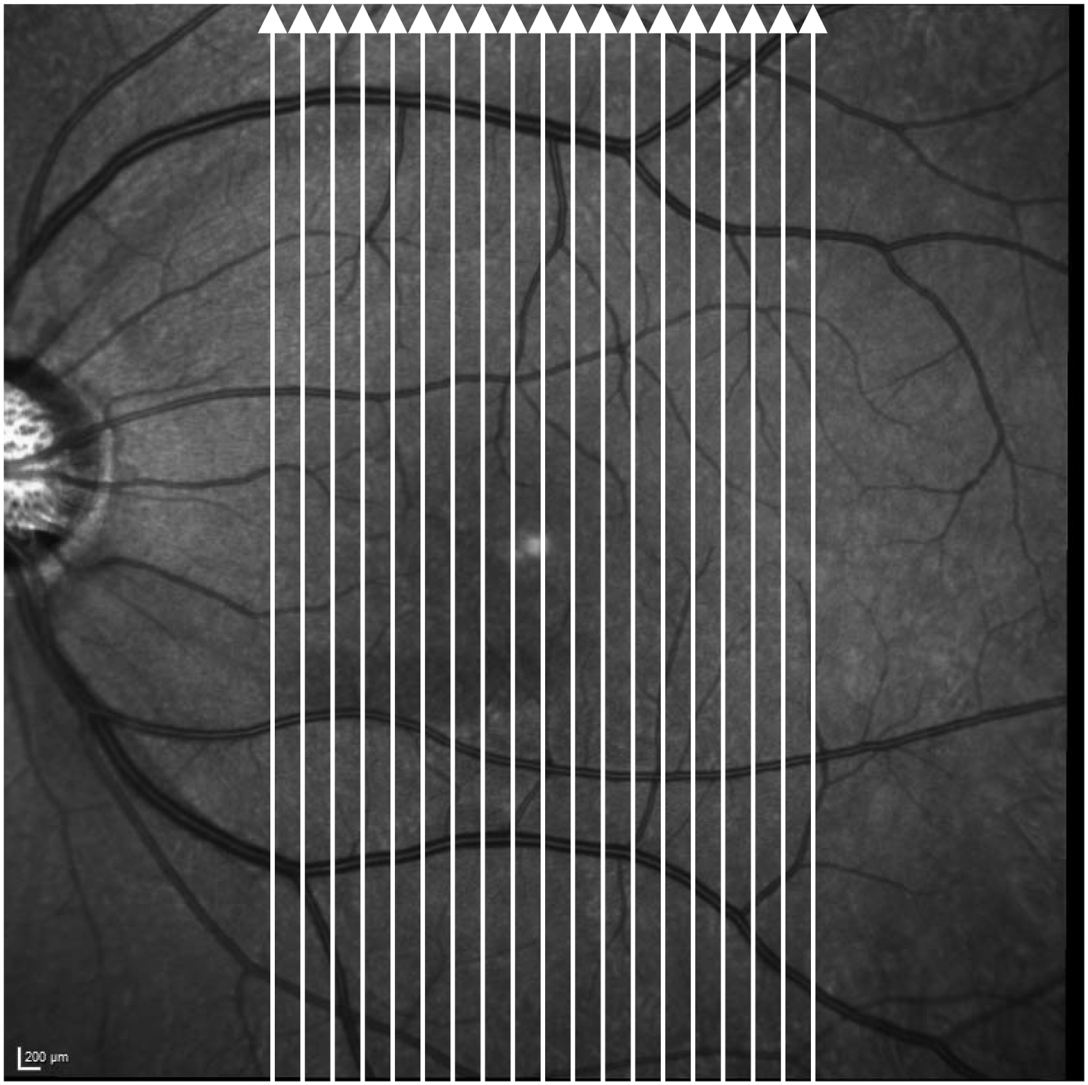
The OCT macular scanning protocol. The protocol consisted of 19 vertical scan lines (white arrows) over macular area. The scan was centered on the fovea and had a height of 30° and a width of 15°. Each of the 19 line scans was obtained by averaging 50 B-scans.

**Fig 3:**
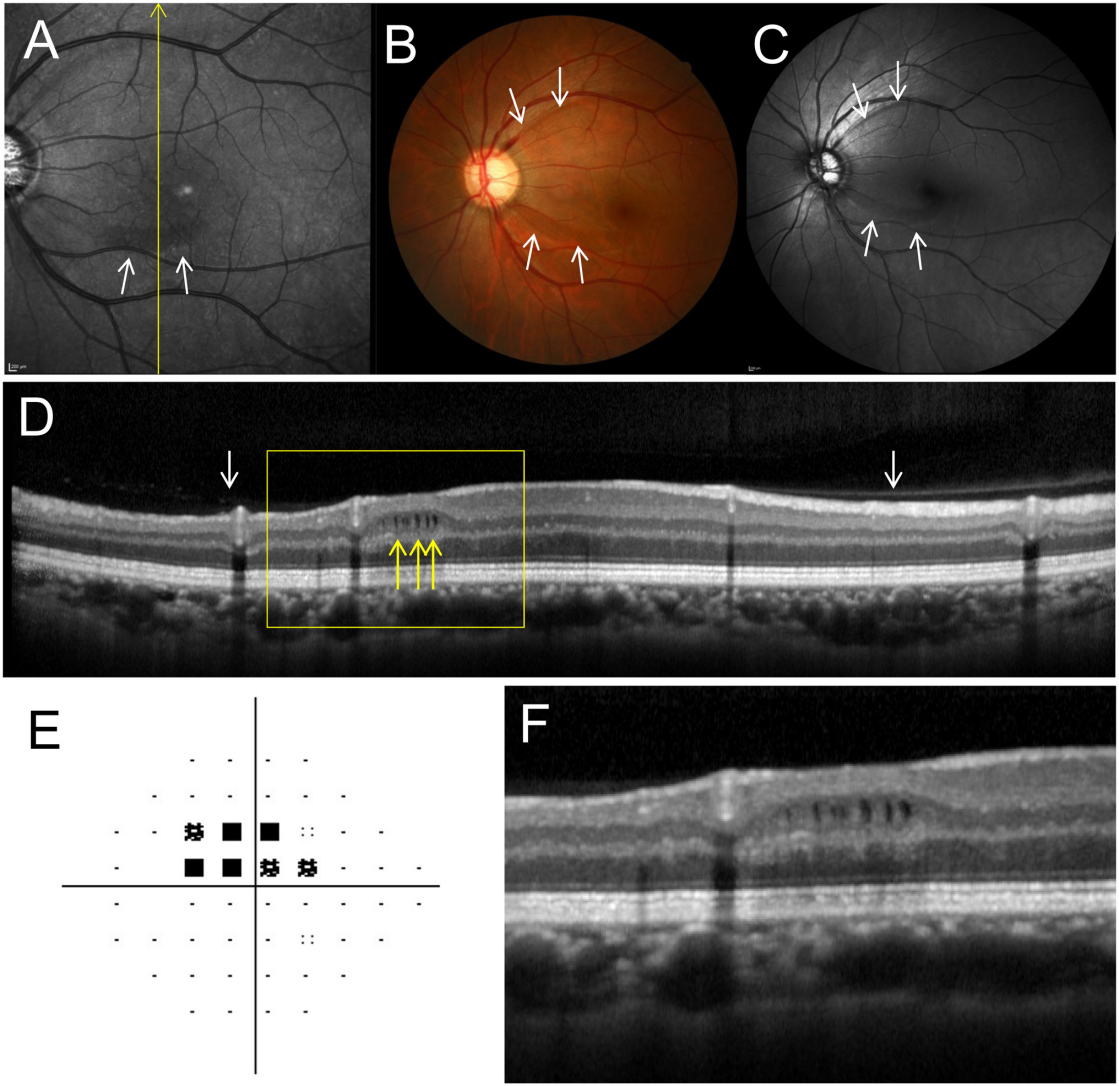
The left fundus of a 44-year-old woman with primary open angle glaucoma and microcystic inner nuclear layer (INL) lesions. **A,** Infrared image shows perimacular hyporeflective patterns in the region with INL microcystic lesions (white arrows). **B, C,** Fundus (**B**) and red-free (**C**) photographs show retinal nerve fiber layer defects (NFLD, white arrows). Disc hemorrhage was also present at the upper NFLD. **D,** A Spectralis optical coherence tomography (OCT) image along the yellow line in **A** shows microcystic INL lesions (yellow arrows). The INL is thicker and the retinal nerve fiber and ganglion cell layers are thinner in the microcystic lesion area. This eye had a partial posterior vitreous detachment (white arrows). **E,** Pattern deviation map from Humphrey Visual Field Analyzer testing (24–2 Swedish interactive threshold algorithm standard program) showing visual field defects. An absolute scotoma at the superonasal test points closest to fixation corresponds to the location of the lower NFLD and microcystic INL lesions. **F,** Magnified OCT image (yellow box in **D**).

**Fig 4:**
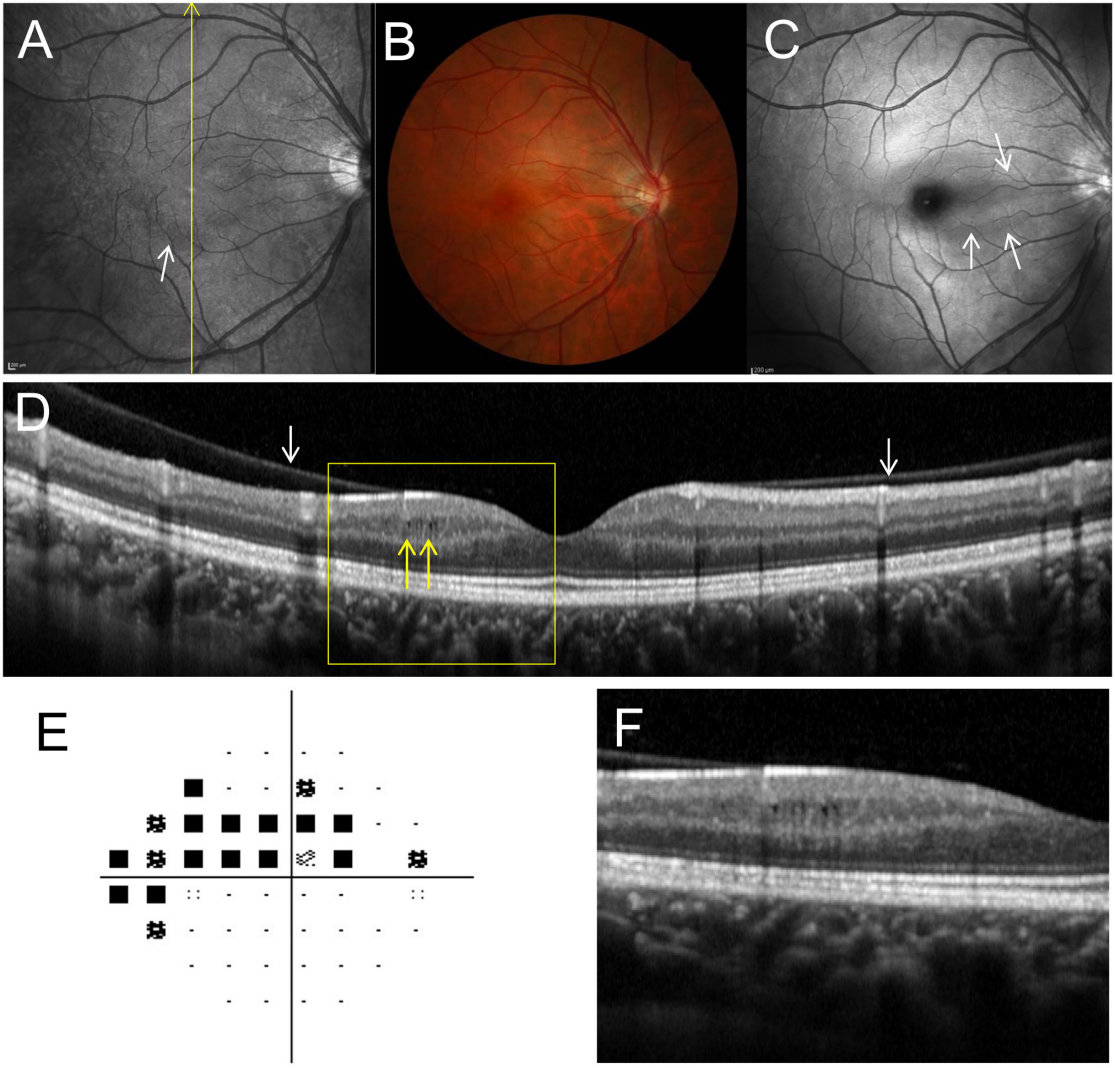
Right fundus of a 29-year-old woman with primary open angle glaucoma and microcystic inner nuclear layer (INL) lesions. **A,** The infrared image shows a perimacular hyporeflective pattern in the region with INL microcystic changes (white arrow). **B,** A fundus photograph shows no evident retinal nerve fiber defect (NFLD). **C**, A red-free photograph shows NFLD in the region of microcystic INL changes (white arrows). **D,** A Spectralis optical coherence tomography (OCT) image along the yellow arrow in **A** shows microcystic INL lesions (yellow arrows). The INL is thicker and the retinal nerve fiber and ganglion cell layers are thinner in the region with microcystic lesions. This eye had a partial posterior vitreous detachment, with the vitreous still attached to the retinal surface above the microcystic lesions (white arrows). **E,** Pattern deviation map from Humphrey Visual Field Analyzer testing (24–2 Swedish interactive threshold algorithm standard program) showed superior visual field defects. An absolute scotoma was present at the superonasal test points closest to the fixation. **F,** A magnified OCT image (yellow box in **D**).

The Spectralis HRA+OCT system was used to scan the macula. Our macular scan protocol scanned a macular area, centered on the fovea, 30° in height and 15° in width. Each retinal scan was made up of 19 vertical line scans (Fig 2), each obtained by averaging 50 B-scans to reduce speckle noise (Figs 3D and 4D). Scanning laser ophthalmoscopy images were also obtained with the Spectralis HRA+OCT to examine perimacular hyporeflective patterns, which have been reported to occur with INL microcystic changes in eyes with optic nerve atrophy [19, 23]. The OCT scans quality was checked as previously reported (no obvious problems; the OCT signal strength > 15 with appropriate averaging of multiple scans; the fundus were well illuminated; the measurement beam placed centrally) [36].

All 19 line scans were manually screened (TH, TA) for the presence of microcystic changes in the INL. To identify the microcystic changes, the same criterion as Gelfand et al. [18] was applied. The microcystic changes were defined as cystic, lacunar areas of hyporeflectivity with clear boundaries, excluding lesions due to speckling artefact, in two or more adjacent B-scans. The evaluators were masked to all other ocular and patient information. If the two evaluators did not agree on INL microcystic changes, all authors reviewed and discussed the scans until a consensus was reached. All NFLDs were diagnosed using fundus photographic and red-free images.

The retinal layers of eyes with microcystic changes were analyzed on SD-OCT images with the longest INL microcystic lesion measurement in the longitudinal direction. The vitreoretinal interface, the RNFL/GCL border, the inner plexiform layer (IPL)/INL border, and the retinal pigment epithelium (RPE) posterior border were determined on SD-OCT after speckle noise reduction, which allows reproducible measurements of retinal layers (based on hand delineation) [3]. The RNFL, GCL+IPL, INL, and outer retinal (from outer plexiform layer to RPE) thickness were measured at the location of the longitudinally longest microcystic lesion and at the opposite location (equidistant site from a horizontal line passing through the foveal center). The segmentation of the macular scans was performed using custom-made software provided by Heidelberg Engineering with manual correction. This segmentation algorithm has been commercially available in Heidelberg Explorer software version 6.0. This analysis was done based on the fact that the retinal structures are highly symmetrical at upper and lower hemispheres in normal eyes [3, 37]. The condition of a PVD was determined and classified, as was done in a previous report [38], with vertical and horizontal Spectralis OCT scans through the fovea in eyes with microcystic lesions.

### Statistical Analysis

All statistical evaluations were performed using commercially available software (SPSS software version 20; IBM, Corp., Armonk, NY). The two-sample t-test or the chi square test was used to compare mean age, gender, axial length, refraction, MD value, MD slope, IOP, and incidence of advanced stage glaucoma between eyes with and without microcystic INL lesions. Wilcoxon signed-rank test was performed to compare the thickness of each retinal layer between areas with and without microcystic lesions in eyes with microcystic lesions. To determine how each retinal layer was affected by microcystic changes, the thickness of each layer at the INL lesion site and at the equidistant, opposite point were compared using the Spearman rank order correlation coefficient. Statistical significance was defined as a *P* value <0.05. Where applicable, data are presented as mean ± standard deviation.

## Results

The medical records of 289 eyes from 153 patients that met the inclusion criteria were reviewed. Of these, 31 eyes were excluded from analyses because of exclusion criteria or unacceptable OCT images. Therefore, 258 eyes from 143 patients were ultimately included in study analyses. The clinical characteristics of included patients are summarized in Table 1. Forty-one eyes were determined to have preperimetric glaucoma and were excluded from the analysis of the characteristics between eyes with or without microcystic INL lesions. A total of 217 eyes (133 patients) were assigned to the glaucoma group, with 95, 58, and 64 eyes having early, moderate, and advanced glaucoma, respectively. The MD slope was analyzed in patients with 4 or more reliable HFA testing results over a minimum follow-up period of two years. This included 184 eyes (120 patients), 13 (13 patients) of which had microcystic changes (Fig 1). Patients with microcystic changes underwent HFA testing an average of 10.9 ± 7.3 times over 54.6 ± 15.1 months. Patients without microcystic changes underwent HFA testing an average of 10.8 ± 6.0 times over 53.2 ± 14.5 months. The mean follow-up period of all subjects was 53.3 ± 14.5 months. One hundred eighty-one eyes from 117 subjects were included as normal control. The clinical characteristics of normal subjects are also summarized in Table 1. None of the normal eyes manifested microcystic INL changes.

**Table 1:** Clinical Characteristics of the Subjects.

Variables	POAG including PPG (n = 258 eyes [143 patients])	Normal (n = 181 eyes [117 subjects])
Age (years)	57.5 ± 11.2 (29 to 79)	59.3 ± 14.7 (22 to 85)
Sex (male/female)	143 (62/81)	117 (61/56)
Spherical equivalent (diopters)	-4.77 ± 3.70 (-12.0 to 2.75)	-2.36 ± 3.23 (-11.50 to 3.00)
Axial length (mm)	25.59 ± 1.68 (21.9 to 29.8)	24.68 ± 1.71 (21.3 to 29.2)
Visual field mean deviation (dB)	-6.46 ± 6.37 (-30.43 to 1.36)	-1.43 ± 1.94 (-8.13 to 2.22)
IOP (mmHg)	15.3 ± 2.2 (9.4 to 24.2)	15.3 ± 2.8 (6.0 to 21.0)
Glaucoma stage		
Advanced, n (%)	95 (36.8%)	
Moderate, n (%)	58 (22.5%)	
Early, n (%)	64 (24.8%)	
Preperimetric, n (%)	41 (15.9%)	

POAG = primary open angle glaucoma, PPG = preperietric glaucoma, IOP = intraocular pressure. Data are presented as mean ± standard deviation or number.

### Patients with Microcystic Changes in the Inner Nuclear Layer

Microcystic INL lesions were identified in 13 (13 patients) of 217 eyes (6.0%) with POAG (excluding PPG). No patient had microcystic INL lesions in both eyes. All eyes with microcystic INL lesions had a BCVA of 20/20 (Snellen equivalent) or better. The demographics and clinical findings of patients with POAG with and without microcystic changes are shown in Table 2. There were no significant differences in sex or age between patients with INL lesions and those without. Additionally, there were no significant differences between these groups in refraction, axial length, IOP, or MD value. However, the percentage of eyes with advanced glaucoma was significantly higher (*P* = 0.013) in eyes with microcystic lesions. The MD slope was also significantly greater (*P* = 0.027) in eyes with microcystic lesions. No eyes in the normal or preperimetric glaucoma groups and only one eye in the early glaucoma stage group had microcystic INL changes.

**Table 2:** Comparison of Subject Characteristics between Primary Open Angle Glaucoma Patients With and Without Microcystic Inner Nuclear Layer Lesion

Variables	Without Microcystic Lesion (n = 204)	With Microcystic Lesion (n = 13)	*P* value
Age (years)	57.7 ± 10.7 (34 to 78)	55.7 ± 15.9 (29 to 79)	0.547
Sex (male/female)	52/68	4/9	0.383
Spherical equivalent (diopters)	-5.00 ± 3.83 (-12.0 to 2.75)	-4.62 ± 3.74 (-10.4 to 1.5)	0.724
Axial length (mm)	25.65 ± 1.68 (21.87 to 29.47)	25.56 ± 2.14 (22.5 to 29.8)	0.854
MD (dB)	-7.52 ± 6.52 (-30.43 to -0.18)	-7.30 ± 4.47 (-15.3 to -1.5)	0.903
MD slope (dB/year)	-0.15 ± 0.44 (-1.98 to 1.84)	-0.43 ± 0.49 (-1.45 to 0.15)	0.027*
Follow up period with HFA (months)	53.2 ± 14.5 (24 to 78)	54.6 ± 15.1 (24 to 67)	0.738
IOP (mmHg)	15.0 ± 2.0 (9.4 to 20.4)	15.0 ± 1.6 (13.0 to 18.6)	0.935
Stage			
Advanced, n (%)	85 (41.7%)	10 (76.9%)	0.013*
Moderate, n (%)	56 (27.5%)	2 (15.4%)	
Early, n (%)	63 (30.9%)	1 (7.7%)	

IOP = intraocular pressure, MD = mean deviation. Data are presented as mean ± standard deviation or number.

*Statistically significant difference (P < 0.05) between the groups by two-sample *t*-test or chi-square test.

### Microcystic Lesion Characteristics

Microcystic INL lesions were observed at all retinal quadrants, but were localized to either the superior or inferior hemisphere in all 13 eyes (3 eyes superior, 10 eyes inferior). Visual field defects were more severe in the superior hemifield than in the inferior hemifield in all 13 eyes. Therefore, microcystic lesions corresponded with more severe visual field defects in 10 of 13 eyes (76.9%). Microcystic changes were not observed at the foveal center, but between 1113 ± 277 μm and 1484 ± 364 away from the foveal center (Figs 3D and 4D). Infrared fundus images revealed perimacular hyporeflective patterns in the area where microcystic lesions were present in 11 of 13 eyes (84.6%; Figs 3A and 4A). Spectralis OCT imaging confirmed that PVD was incomplete (paramacular PVD) in 10 eyes (76.9%) (Figs 3D and 4D). The remaining 3 eyes (23.1%) had complete PVDs.

### Time Course of Microcystic Changes in the Inner Nuclear Layer

**Fig 5:**
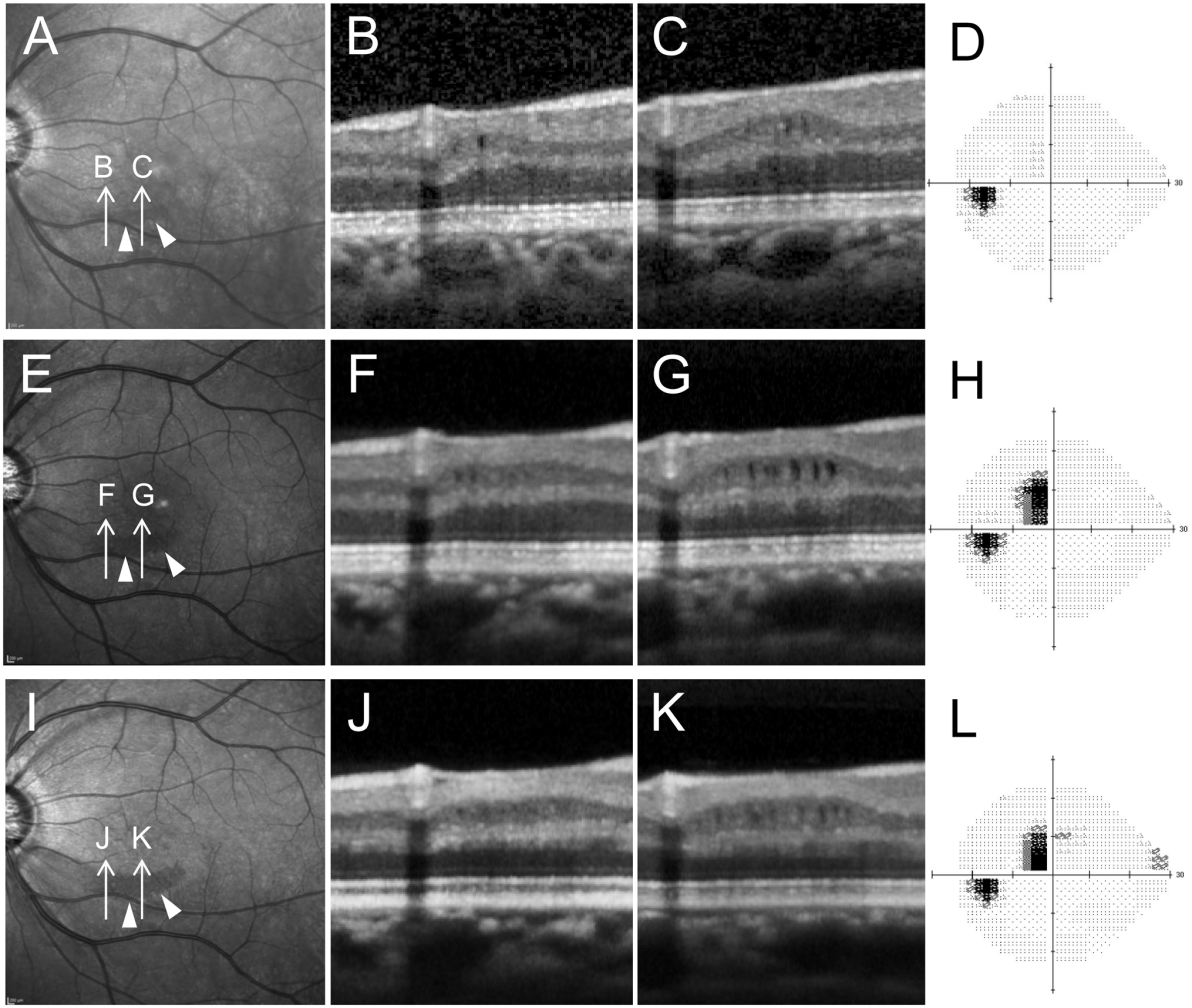
Changes over time in an eye with microcystic inner nuclear layer (INL). Changes appearing before significant visual field damage occurred. **A-D, E-H** and **I-L** show images and testing obtained in December of 2008, 2012, and 2013, respectively. **A, E, I,** Infrared images showed perimacular hyporeflective patterns (arrow heads) becoming more obvious over time. **B, C, F, G, J, K,** Spectralis OCT images oriented along arrows in **A, E** and **I**. **D, H** and **L,** Standard automated perimetry testing results (Humphrey Visual Field Analyzer, 24–2 Swedish interactive threshold algorithm standard program gray scale). Subtle microcystic changes were observed in INL (**B, C**). It was noticed that localized thinning of retinal nerve fiber layer and ganglion cell layer existed though no severe visual field defects were present (**B, C** and **D**). Microcystic changes became more distinct as visual field defects progressed (**F, G** and **H**). Microcystic lesion changes became less apparent (**F, G, J** and **K**) while the visual field remained stable (**H, L**).

Eleven of 13 eyes (84.6%) with microcystic INL lesions had undergone more than two Spectralis OCT examinations (mean of 10.6 ± 7.4 times). Microcystic changes increased in 2 of 13 eyes (15.4%) over a 58.0 ± 2.8 month observation period (Fig 5) and remained stable or became indistinct in 9 eyes over a 33.1 ± 20.4 month observation period. In one eye, microcystic changes appeared before severe visual field defects (Fig 5, same case as shown in Fig 3).

### Correlation between Microcystic Inner Nuclear Layer Change and Retinal Nerve Fiber Layer Defects

**Fig 6:**
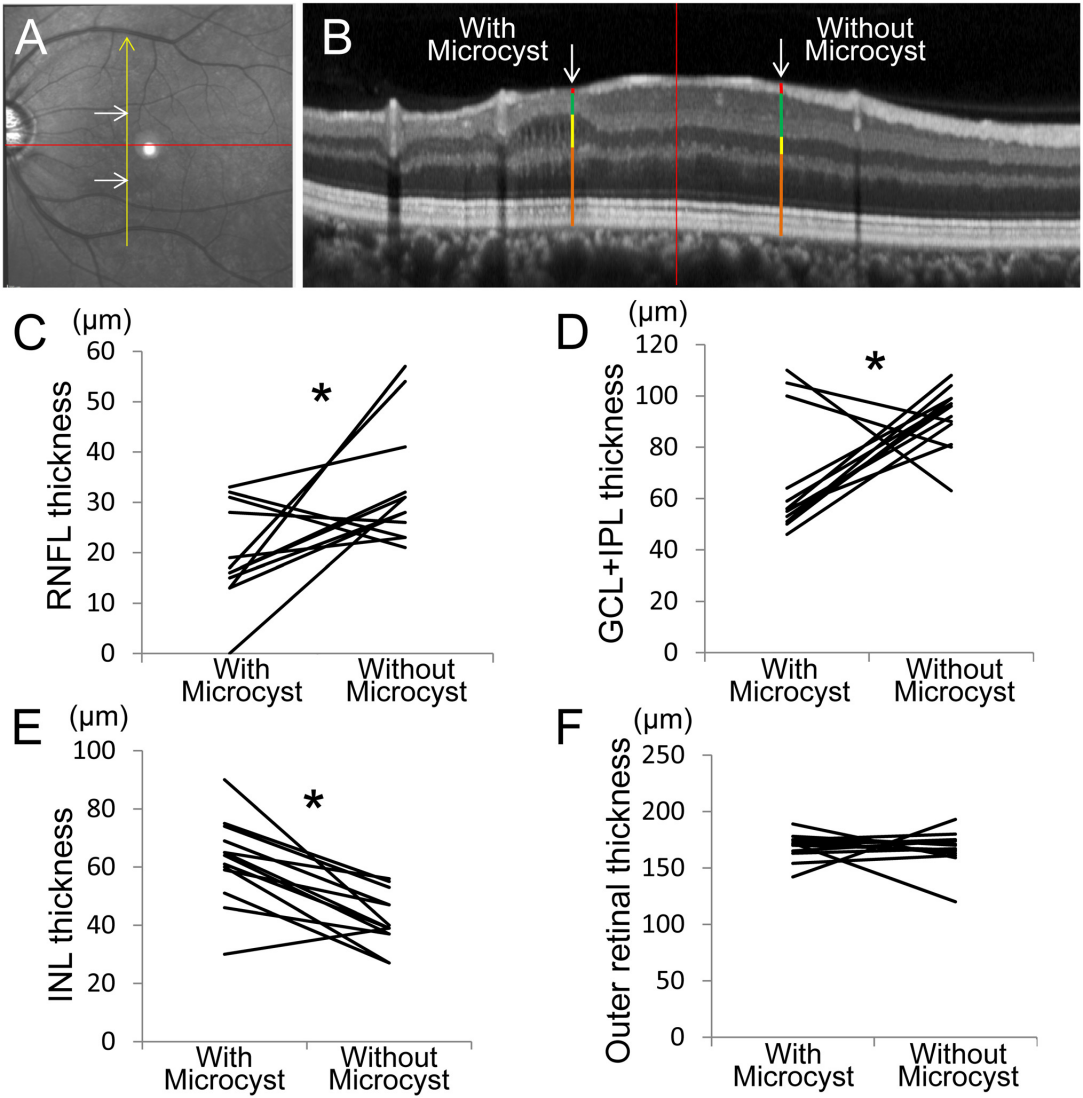
Comparison of retinal nerve fiber layer (RNFL), ganglion cell complex plus inner plexiform layer (GCC+IPL), inner nuclear layer (INL), and outer retinal thickness between at sites with and without microcystic changes. Measurements were taken at the opposite and equidistant site from a horizontal line passing through the foveal center (red line in **A**) for sites with and without INL lesions. **A,** An infrared fundus image shows measurement points (white arrows). **B,** A Spectralis optical coherence tomography (OCT) image along the yellow arrow in **A**. The RNFL (red bars), GCL+IPL (green bars), INL (yellow bars) and outer retinal thickness (brown bars) measured at sites with and without microcystic lesions (white arrows) and are shown in parts in **C, D, E** and **F**, respectively.

Apparent NFLDs were observed in 11 of 13 eyes (84.6%) with microcystic changes. Eight of these eyes (72.7%) showed microcystic changes in the same area as the NFLD (Figs 3 and 4). Areas with microcystic changes also had RNFL thinning in 12 of 13 eyes (92.3%), as visible on Spectralis OCT images. Retinal layer thickness changes were quantified by measuring RNFL, GCL+IPL, INL, and the outer retinal (from outer plexiform layer to RPE) thickness in the OCT scan showing the longest longitudinal lesion dimension. The measurements were then compared to those taken at the opposite and equidistant location from a horizontal line passing through the foveal center (Fig 6A and 6B). This comparative method was chosen because retinal layer thickness is highly symmetrical between retinal hemispheres in normal eyes [3, 37]. The RNFL and GCL+IPL were significantly thinner on the side with microcystic changes than on the side without changes (RNFL: 19.2 ± 16.7 μm vs. 33.6 ± 11.6 μm, *P* = 0.013; GCL+IPL: 65.8 ± 22.8 μm vs. 91.9 ± 11.8 μm, *P* = 0.023; Fig 6C and 6D). However, the INL was significantly thicker on the side with microcystic changes (62.2 ± 14.7 μm) than on the side without changes (41.8 ± 9.4 μm, *P* = 0.002, Fig 6E). Outer retinal thickness was not significantly different between the affected (169.1 ± 11.5 μm) and unaffected (166.5 ± 16.6 μm) locations (*P* = 0.649, Fig 6F).

The difference in INL thickness between affected and unaffected locations (20.5 ± 13.9 μm, Fig 6E) was significantly correlated with RNFL thickness differences (-14.5 ± 16.7 μm, Fig 6C; ρ = -0.65, *P* = 0.015) and GCL+IPL thickness differences (-26.1 ± 32.1 μm, Fig 6D; ρ = -0.577, *P* = 0.039). However, INL thickness differences were not correlated with ganglion cell complex thickness differences (-44.5 ± 48.1 μm; *P* = 0.071) and outer retinal thickness differences (2.6 ± 23.4 μm, Fig 6F; *P* = 0.893).

## Discussion

The main finding of this study was that microcystic INL lesions were observed on SD-OCT images in 6.0% eyes with POAG (5.0% if including eyes with preperimetric glaucoma), and the microcystic change location was significantly correlated with that of NFLDs, INL thickening, and RNFL/GCL thinning. Additionally, microcystic lesions were more frequently observed in eyes with advanced stage glaucoma and were significantly associated with worse MD slope of visual field.

Microcystic INL lesions have been observed in eyes with various optic nerve diseases [18–27]. These microcystic lesions were characterized by an INL hyporeflectivity on OCT B-scans and infrared fundus images. These findings have been reported in about 5% of patients with MS [18, 27], a similar incidence to that found in the current study with glaucomatous eyes. Microcystic lesions were equally distributed among the retinal quadrants in MS patients [18], but were mainly observed in the inferior hemisphere in glaucomatous eyes (10 of 13 eyes). This difference in lesion location could result from differences between the two kinds of optic neuropathy in optic nerve damage location. Glaucomatous optic neuropathy occurs, and tends to be more severe, in the inferior quadrants [39], and, based on the results of the current study, microcystic changes are more likely to appear in locations corresponding to visual field defects.

It has been reported that microcystic changes are associated with lower visual acuity in MS patients [18, 27]. In the current study, microcystic changes were more likely to be observed in eyes with advanced stage glaucoma (10 of 13 eyes) (Table 2). However, there was no difference in MD value between eyes with POAG with microcystic changes and eyes with POAG and no microcystic changes. Interestingly, there was no eye which manifested microcystic changes with MD value worse than -15.3 dB. There was a significant difference in MD slope between eyes with and without microcystic lesions in eyes with POAG. These results suggest that the microcystic INL lesion is more likely to appear in eyes with rapid progression and without total damage of RNFL and GCL. It was previously reported that younger patients are significantly more likely to develop microcystic changes in eyes with optic neuropathy (excluding glaucoma) [34]. However, we found no significant age difference between patients with and without microcystic changes.

The mechanism by which microcystic changes occur in glaucomatous eyes remains unclear, but several theories have been suggested in patients with other types of optic neuropathy including MS. These include inflammation induced blood-retinal barrier disruption [18, 24, 27], Muller cell dysfunction [21], retrograde trans-synaptic degeneration [25, 34], vitreous traction [26], and combined of the trans-synaptic degeneration and vitreous traction [40].

We believe that microcystic-like changes are non-specific, non-inflammatory in nature, and are affected, at least in part, by mechanical traction [25, 26, 40]. Partial PVD, the ILM, and retinal arteriole stiffness have all been implicated in causing myopic schisis, which is characterized by an inner retinal separation [32, 33]. Eyes with obvious vitreoretinal abnormalities (e.g., ERM and vitreoretinal traction syndrome) were excluded from this study. Only a few eyes had complete PVDs in our study. The vitreous remained attached in the area of microcystic lesions in 76.9% of eyes with microcystic INL lesions (Figs 3D and 4D). It should be noted that our patients actually had a lower incidence of complete PVD (23.1%) than that of normal eyes in patients of the same age (48.4%, calculated based on consideration for their ages) [38]. Furthermore, we discovered that microcystic lesions generally begin developing near arterioles that are close to areas of RNFL and GCL thinning. Fig 5 shows a case in which retinal thickness was preserved by ILM and retinal arteriole stiffness. Xin D et al. [41] has reported microcystic changes in the RNFL in glaucomatous eyes and proposed mechanical force as the cause since they were located adjacent to blood vessels and associated with arcuate defects. On the other hand, Brandt et al. [42] has shown that vitreous traction theory is unlikely to be a causative factor in macular microcyst formation using a mechanical model. Green et al. [43] was also opposite to vitreous traction theory and stated that there were some cases with complete PVDs with microcystic changes. Indeed, there were also some cases with complete PVDs in our cases. However, we believe that this fact cannot completely deny the mechanical theory. In the current study, the INL was thicker at the site of the microcystic changes compared to the opposite and equidistant site from a horizontal line passing through the foveal center in the eyes with microcystic lesions. This result is consistent with the previous reports showing thickening of INL and thinning of RNFL and GCL+IPL in NMO or MS patients [22, 23, 26, 27]. We hypothesize that ILM and retinal arteriole stiffness themselves may act as scaffolds of retinal thickness and mechanical forces to thicken INL with thinning of RNFL and GCL, which could cause schisis-like microcystic INL changes even after PVDs were occurred.

The retrograde trans-synaptic degeneration mechanism is another theory proposed as a cause of microcystic changes [25, 34, 44]. It has been reported that trans–synaptic axonal degeneration occurs in the visual pathway of patients with MS [45] and microcystic INL changes in OCT images are very similar to histological findings following retrograde trans-synaptic degeneration from a tumor of the optic nerve [40, 46]. Green et al. [47] histologically demonstrated that INL was a prominent site of atrophy in MS eyes. These previous reports support the retrograde trans-synaptic degeneration theory in microcystic INL lesion formation. However, this theory is not likely to be the only pathogenic mechanism in our glaucoma patients because microcystic changes would occur much more frequently in advanced stages of glaucoma if this were the only cause. Additionally, microcystic changes appeared in one eye when only a subtle visual field defect was present and occurred with glaucoma progression. This implies that retrograde trans-synaptic degeneration was not likely the only cause of microcystic INL lesion development.

The fact that there was no eye which manifested microcystic changes with MD value worse than -15.3 dB might imply the possibility that microcystic changes tend to occur with localized RNFL and GCL thinning rather than widespread RNFL and GCL thinning with severely damaged visual field. This fact means that microcystic INL lesion formation could not be explained by only retrograde trans-synaptic degeneration theory. However, we cannot exclude the possibility that INL atrophy caused by trans-synaptic degeneration may accelerate mechanical generation of INL microcystic changes together with the INL thickening, the thinning of RNFL and GCL and the stiffness of ILM and retinal arteriole.

The proposed mechanism of inflammation-induced blood retinal barrier disruption seems to be unlikely in glaucomatous cases because fluorescein leakage is not associated with glaucoma [19, 26]. However, it is possible that there are several mechanisms to cause the microcystic INL changes. Blood retinal barrier disruption may affect microcystic INL changes in NMO cases, which are associated with inflammation [48, 49] and retinal vascular changes [50]. We cannot deny that the pathogenic mechanism of microcystic INL changes is multifactorial even in glaucoma patients.

The microcystic changes were associated with faster progression of visual field defect and were not observed in the totally damaged eyes. There is a possibility that the eyes with progressive visual field defects and focal thinning of RNFL and GCL tend to cause mechanical inner retinal separation and microcystic INL changes under the influence of mechanical traction including ILM stiffness. Saidha et al. has reported that the INL thickening was related to disease progression and relapses in MS patients [27]. Microcystic INL thicknening might offer information of progressive and focal damage in optic neuropathy including glaucoma.

It is known that SD-OCT allows reproducible measurements of GCC and macular GCC thickness to be made and that these are useful in diagnosing glaucoma [1, 3–6, 10]. It should be noted that microcystic INL lesions may affect total retinal thickness by increasing INL thickness, although total retinal thickness is not generally used for diagnosing and monitoring glaucoma. Furthermore, some eyes with microcystic changes had OCT measurements with obvious GCC autosegmentation errors in areas with microcystic INL lesions (data not shown), even though it was not difficult to manually delineate the GCC. Therefore, physicians should be aware that microcystic lesions may affect automated GCC measurements, depending on the segmentation algorithm used.

This study had several limitations. First, it is a retrospective study and included a relatively high proportion of myopic patients. We chose to include highly myopic eyes in our study because our glaucoma service has many highly myopic glaucoma patients. Therefore, excluding highly myopic patients would have limited our patient numbers and interfered with examining the effect of myopia on microcystic changes. Interestingly, myopia did not statistically affect microcystic lesion development or location. Second, we used a Spectralis OCT macular scan protocol that consisted of 19 vertical line scans to identify microcystic changes [10]. Although this method, in which 50 individual B-scans were averaged to obtain each of the 19 line scans, gave us high quality OCT images, too many averaging of B-scans might lead reduced detection of minute microcystic changes. This scanning protocol is, furthermore, thought to cover almost the entire macula region, but subtle microcystic changes outside of the scanning region could have been overlooked. We defined the microcystic lesions when identified in two or more adjacent B-scans to avoid false-positive cases. This method possibly underestimated the number of eyes with microcystic lesions. Third, we did not examine associations between fluorescein leakage and microcystic INL changes because fluorescein angiography had not been routinely performed in our patients. However, previous studies did not find leakage in any eye with microcystic INL lesions associated with glaucoma or other optic nerve diseases [19, 26].

In conclusion, microcystic INL changes are more frequently observed in eyes with advanced stages of glaucoma without total damage and are associated with progressive visual field defects and localized thinning of RNFL and GCL. Microcystic INL changes may indicate focal and progressive damage in glaucoma.

## Supporting Information

S1 File Specific data of each group. Detail data of POAG group and normal group, and the subjects with microcystic inner nuclear layer lesions.
(XLSX)
